# Bias Voltage Dependence of Sensing Characteristics in Tunneling Magnetoresistance Sensors

**DOI:** 10.3390/s21072495

**Published:** 2021-04-03

**Authors:** Piotr Wiśniowski, Maciej Nawrocki, Jerzy Wrona, Susana Cardoso, Paulo. P. Freitas

**Affiliations:** 1 Institute of Electronics, AGH University of Science and Technology, 30-059 Krakow, Poland; mnawrocki@student.agh.edu.pl; 2 Singulus Technologies AG, 63796 Kahl am Main, Germany; jerzy.wrona@singulus.de; 3 INESC Microsystems and Nanotechnologies, INESC-MN, and IN, 1000-029 Lisbon, Portugal; scardoso@inesc-mn.pt (S.C.); pfreitas@inesc-mn.pt (P.P.F.); 4 Physics Department, Instituto Superior Tecnico, Universidade de Lisboa, 1600-276 Lisbon, Portugal

**Keywords:** magnetic field sensors, tunneling magnetoresisatnce sensors, sensing characteristics, bias voltage effect on sensing characteristics

## Abstract

One of the characteristic features of tunneling magnetoresistance (TMR) sensors is a strong influence of bias voltage on tunneling current. Since fundamental sensing characteristics of the sensors are primarily determined by the tunneling current, the bias voltage should impact these characteristics. Previous research has indeed showed the influence of the bias voltage on the magnetic field detection and sensitivity. However, the effect has not been investigated for nonlinearity and hysteresis and the influence of bias voltage polarity has not yet been addressed. Therefore, this paper systematically investigates the dependence of field sensitivity, nonlinearity, hysteresis and magnetic field detection of CoFeB/MgO/CoFeB-based magnetoresistance sensors on bias voltage magnitude and polarity. The sensitivity and field detection of all sensors improved significantly with the bias, whereas the nonlinearity and hysteresis deteriorated. The sensitivity increased considerably (up to 32 times) and linearly with bias up to 0.6 V. The field detection also decreased substantially (up 3.9 times) with bias and exhibited the minimum values for the same magnitude under both polarities. Significant and linear increases with bias were also observed for nonlinearity (up to 26 times) and hysteresis (up to 33 times). Moreover, not only the voltage magnitude but also the polarity had a significant effect on the sensing characteristics. This significant, linear and simultaneous effect of improvement and deterioration of the sensing characteristics with bias indicates that both bias voltage magnitude and polarity are key factors in the control and modification of these characteristics.

## 1. Introduction

The tunneling magnetoresistance (TMR) sensors based on a CoFeB/MgO/CoFeB structure exhibit unique design properties and high-performance sensing capabilities. They can be designed with the resistance of the arbitrary value (Ohm-MOhm) by changing the thickness of the tunneling barrier and the active size down to nanometers [1]. The sensors can be easily integrated with CMOS technology for a compact design and improved reliability [2]. Importantly, due to a large tunneling magnetoresistance ratio (TMR) [3,4] the sensors provide high field sensitivity [2]. Moreover, sensor design based on perpendicular magnetic anisotropy in the sensing layer enables a simple and effective modification of the magnetic field sensing range and field sensitivity by means of thickness [5] and voltage-induced modulation of anisotropy [6]. The sensors have also realized magnetic field detection levels beyond the magnetic noise-sensitivity scaling limit [7]. These features and properties make TMR sensors an important class of solid-state magnetic field sensors that can be designed to sense magnetic fields in a wide range, with high sensitivity, high spatial resolution (down to nm) and ultra-low power consumption. Importantly, the sensors can be used to sense not only the magnetic field but also to indirectly sense magnetic nanoparticles, electric current, rotation, and angle [8,9,10].

One of the characteristic features of tunneling magnetoresistance sensors is a strong influence of bias voltage on the spin-dependent tunneling current [11]. The increase in the current along with bias voltage causes a reduction in device resistance and consequently the TMR ratio [12,13,14,15,16], which is defined as the ratio of resistance in antiparallel (R_ap_) and parallel (R_p_) magnetization configurations of the ferromagnetic electrodes (TMR = (R_ap_ − R_p_)/R_p_). The bias voltage also strongly influences both frequency-dependent [17] and frequency-independent noise [18]. Consequently, the bias voltage is important for TMR sensors because their sensing characteristics are derived from the tunneling current. 

Sensing characteristics of TMR sensors such as field sensitivity, nonlinearity, hysteresis and magnetic field detection are primarily determined by the tunneling current and its fluctuation. The field sensitivity is determined from sensors’ transfer curves (current versus magnetic field, I-B), expressed as dI/dB. Nonlinearity is typically determined as the deviation of the current (δI) from a linear fit to the I-B curve. The hysteresis is determined as the difference (ΔI) between the currents for increasing and decreasing magnetic fields. The field detection is typically determined as a ratio of sensor noise to the field sensitivity or is measured directly. The derivation of the sensing characteristics from the tunneling current and its strong change with the bias voltage indicate the crucial role of the bias voltage in modifying and controlling these characteristics.

Previous research on the influence of bias voltage on TMR sensor characteristics is limited to field detection [17,19], sensitivity [20] and a special type of sensor based on a voltage-controlled magnetic anisotropy design [7]. A change in field detection with bias voltage has been reported for MgO-based TMR devices with square transfer curves [19] and for transfer curves with hysteresis [18]. The investigated field detection was calculated from the measured noise and bias voltage-dependence of the TMR ratio. The devices showed different behaviors in terms of of the field detection with bias voltage, depending on the frequency range. At frequencies where 1/f noise dominates, the field detection deteriorated with increasing voltage and showed no saturation in the measured range (up to 1 V). In contrast, at frequencies where white noise dominates, the field detection improved with bias voltage in a low range and then at higher saturation. The effect of bias voltage on sensitivity was reported for MgO-based TMR sensors with negligible hysteresis [20]. The sensors showed an increase in the sensitivity and saturation around 0.7 V. The reported results indicate a substantial change in the field detection and sensitivity with the bias voltage. However, there is a lack of systematic investigation of the bias dependence of the sensing characteristics, including the effect the voltage polarity. 

Therefore, this work systematically investigates the influence of bias voltage polarity and magnitude on field sensitivity, nonlinearity, hysteresis and magnetic field detection of CoFeB/MgO/CoFeB-based tunneling magnetoresistance sensors. It focuses on sensors designed with the same properties but significantly different field sensitivities of 2.7 V/T, 45 V/T and 127 V/T. It shows that the field sensitivity and magnetic field detection of all sensors improved significantly with the bias, whereas the nonlinearity and hysteresis deteriorated. The sensitivity increased strongly (up to 32 times) and at a high rate (up to 266 V/T/V) and linearly with bias voltage up to 0.6 V. The field detection performance also declined significantly (up 3.9 times) with bias and exhibited the minimum values for the same magnitude of 0.5 V under both polarities for all sensors. A strong increase with bias was also observed for nonlinearity (up to 26 times) and hysteresis (up to 33 times). They also increased linearly with bias up to a significant magnitude (about 0.6 V). Moreover, the voltage magnitude, as well as the polarity, had a considerable influence on the sensing characteristics.

## 2. Sensor Fabrication and Characterization

**Figure 1:**
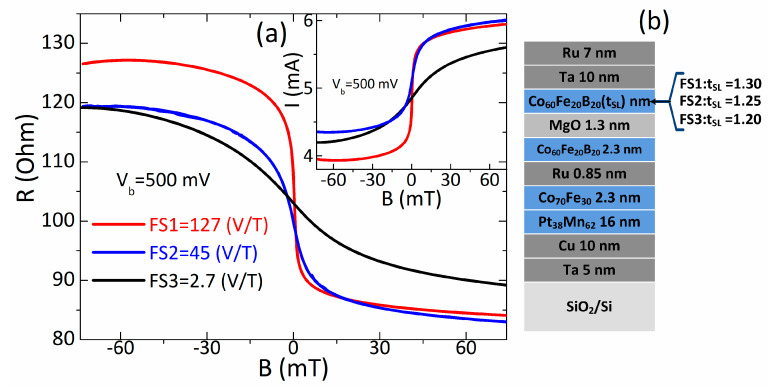
Transfer curves (current versus magnetic field (I-B), resistance versus magnetic field R-B,) of the sensors designed to have high, medium and low sensitivity (**a**). Material structure of the sensors (**b**).

To investigate the influence of bias voltage on the sensing characteristics, we fabricated sensors with the same properties but with significantly different field sensitivities (Figure 1a). We designed sensors using perpendicular magnetic anisotropy in the sensing layer (SL) [5]. To achieve sensors with different field sensitivities (FS), such that FS1 > FS2 > FS3, we used sensing layers with thicknesses (t_SL_) as follows: FS1:t_SL_ = 1.30 nm; FS2:t_SL_ = 1.25 nm; and FS3:t_SL_ = 1.20 nm. The thinner sensing layer induced stronger perpendicular anisotropy, which resulted in larger saturation field of the layer (saturation field ~1/t_SL_). The larger saturation field caused the lower slope of the transfer curve, thus lower sensitivity. Moreover, the thinning of the sensing layer reduces the TMR ratio. Both of these changes contributed to the modification of the sensitivity through the sensing layer thickness. More details on the sensor design can be found in [5,18,19,21,22].

The sensor material stacks (Figure 1b) were deposited on 6-in. wafers using a TIMARIS sputtering system at Singulus AG using a linear dynamic deposition technique (Patent US 7,799,179). The metallic layers were deposited by DC magnetron sputtering and MgO by radio frequency RF magnetron sputtering. The linear dynamic deposition technique guarantees the homogeneity and thickness of the layers. The structure and thickness of the layers were verified during the optimization of the deposition process at Singulus AG. The exemplary transmission electron microscopy images of similar material structures deposited by the TIMARIS system were presented in [23]. 

The sensors were fabricated at INESC-MN using a laser lithography microfabrication process. The 6-in. wafers were diced into 1 in. × 1 in. wafers. The 1-in. wafers were patterned using direct-writing laser lithography and ion beam milling. All sensors were patterned into circular shapes with a diameter of 60 µm. The patterned 1 × 1-in wafers were annealed in a high vacuum at 340 °C for 1 h in a magnetic field of 0.5 T. The resistance of the sensors at zero external magnetic field was about 100 Ohm.

In investigating the dependence of the sensing characteristics on the bias voltage, we focused on field sensitivity, nonlinearity, hysteresis and magnetic field detection. The field sensitivity (FS) in unit V/T was calculated from the current versus magnetic field (I-B) curves as FS = (dI/dB)R for zero magnetic field. The nonlinearity was determined from fitting the experimental I-B transfer curves (inset of Figure 1) to a linear function. Based on the deviations of the current (δI) from the linear fits, we obtained the maximal nonlinearity (NL_max_). The maximal hysteresis (HR_max_) was determined as the maximal difference σI between the current for the increasing field and the current for the decreasing field. These sensing characteristics constitute a set of important properties that indicate sensor performance and potential applications.

We measured I-B transfer curves and the field detection of the sensors to investigate the influence of bias voltage on the sensing characteristics. The transfer curves were measured using a DC four-probe setup with Helmholtz coils as a magnetic field source and source measure unit (Keithley 2636A) in constant voltage mode under positive and negative biases. The magnetic field detection performance was measured in a shielded box, containing a battery-powered low-noise amplifier (Femto DLPVA 100), voltage bias circuit, and Helmholtz coils generating a magnetic field. The field detection was directly measured by applying AC magnetic fields with frequencies of 10 Hz, 10 kHz and 80 kHz to the sensors and recording the sensors’ signals using the spectrum analyzer technique. The low- and high-frequency field detection performance was determined as the magnetic field at which the sensor signal reached its noise level.

## 3. Bias Dependence of Sensing Characteristics

### 3.1. Field Sensitivity 

**Figure 2:**
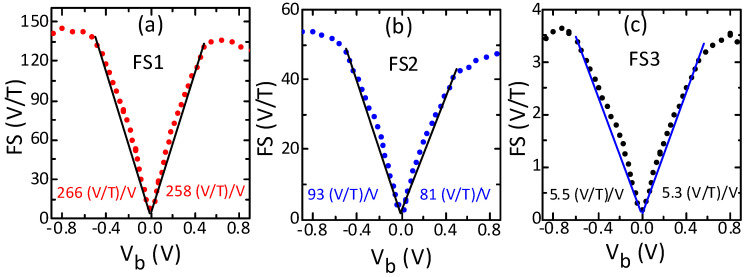
Bias voltage influence on the field sensitivity (FS) of the sensors with high (**a**), medium (**b**) and low (**c**) sensitivity. The sensitivity increases linearly up to a certain bias but with higher rate under negative polarity. It also reaches a higher maximum value under negative polarity. The linear ranges of sensitivity were determined by fitting the linear model to the measured data using the least squares method. Solid lines represent the linear fits and the dotted lines are the experimental results.

All sensors showed a rapid and linear increase in field sensitivity with bias voltage up to a certain voltage magnitude and higher rates and maximum values under negative polarity (Figure 2). In the bias voltage range up to about 0.6 V, the field sensitivity increased approximately linearly for all sensors. However, the increase was higher under negative bias (12 V/T/V) than under positive bias, especially for sensors FS1 and FS2. Above this bias, the sensitivity increase was slow and non-linear, reaching a maximum value in the range of 0.75 V to 0.9 V. The field sensitivity increased from the minimum to the maximum by a factor of 17, 20 and 32 for sensors FS1, FS2 and FS3, respectively. The maximum values of field sensitivity were again higher under negative bias and especially for higher-sensitivity sensors (FS1, FS2). At higher bias, the sensitivity declined slightly, except for the sensor with low sensitivity (FS3), where it declined considerably. The significant improvement (increase) in field sensitivity with bias magnitude implies that the reduction of TMR ratio [9,10] typically observed for these devices with bias voltage was lower than the current increase. Importantly, the rate of increase and maximal values of the field sensitivity were higher under negative bias voltage. Moreover, the field sensitivity increased linearly up to a significant bias (~0.6 V). The behavior of the field sensitivity with bias voltage shows that the applying of proper bias magnitude and polarity enables significant improvement and linear modification of the field sensitivity of the sensors.

### 3.2. Nonlinearity

**Figure 3:**
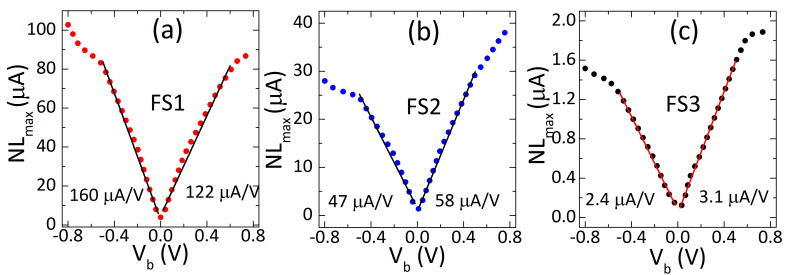
Influence of bias voltage on maximal nonlinearity (NLmax) of high (**a**), medium (**b**) and low (**c**) sensitivity sensors. The nonlinearity is expressed as the maximal residual of linear fit to the transfer curves of the sensors. The nonlinearity increases approximately linearly up to a certain bias, but with a higher rate under positive polarity. It also reaches a higher maximum value under negative polarity. The linear ranges of the nonlinearity were determined by fitting the linear model to the measured data using the least squares method. Solid lines represent the linear fits and the dotted lines are the experimental results.

The maximal nonlinearity (NL_max_) of the sensors increased strongly with bias magnitude, but at higher rate for positive bias polarity than negative (Figure 3). The change in the maximum nonlinearity with bias was correlated with field sensitivity—the higher the sensitivity (Figure 2), the higher the rate of increase of the nonlinearity with bias (FS1: 160 μA/V to FS3: 2.4 μA/V). The rate of increase was lower by a factor of 1.3 under negative bias, except for the highest sensitivity sensor (FS1), where it was lower under positive bias. The nonlinearity changed from the minimum to the maximum by a factor of 26, 20 and 10 for FS1, FS2 and FS3, sensors, respectively. Moreover, it increased approximately linearly with the bias up to 0.4 V–0.5 V (Figure 3). Above this bias voltage, the increase in the nonlinearity was not monotonic. Again, the sensors under negative bias showed lower nonlinearity values, except for sensor FS1. In contrast to the sensitivity, the nonlinearity deteriorated (increased) with the bias of both polarities. It increased strongly, but the increase rate and the highest values of the nonlinearity were significantly different for negative and positive bias. These results indicate the deterioration of nonlinearity regardless of bias polarity, but at a lower rate under negative polarity for most sensors.

### 3.3. Hysteresis

**Figure 4:**
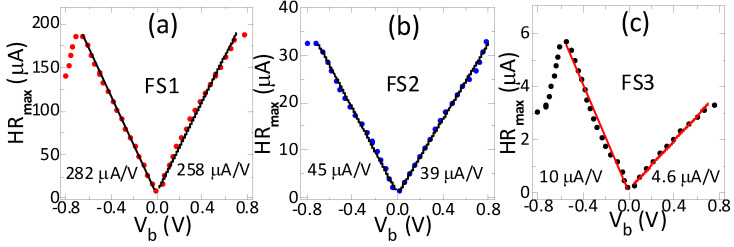
Effect of bias voltage on hysteresis (HR_max_) of sensors with high (**a**), medium (**b**) and low (**c**) sensitivity. The hysteresis considerably changed with bias magnitude and polarity and was lower under positive negative polarity. The linear ranges of the hysteresis were determined by fitting the linear model to the measured data using the least squares method. Solid lines represent the linear fits and the dotted lines are the experimental results.

The maximal hysteresis (HR_max_) increased linearly and very strongly with bias and at a higher rate under negative bias (Figure 4). Similar to the nonlinearity, the hysteresis was the highest for the highest sensitivity sensor (FS1) and the lowest for the lowest sensitivity sensor (FS3). The hysteresis increased approximately linearly up to 0.6 V–0.75 V and changed by a factor of about 17 to 33 between the minimum and maximum bias. The increase rate was higher under negative polarity by a factor of 1.1 for sensors FS1 and FS2 and by a factor of 2.1 for FS3. Under negative polarity, hysteresis was saturated above 0.6–0.75 V and then declined most significantly (by about two times) for sensor FS3. In contrast, the hysteresis under positive polarity increased with bias voltage without saturation. The maximum hysteresis values were slightly larger under negative bias for all sensors, but for sensor FS3 the maximum value under positive polarity was higher by a factor of 1.9.

The increase rate and the highest hysteresis values were lower under positive bias. Moreover, the hysteresis increased linearly up to a significant bias, as did the field sensitivity and nonlinearity. These results indicate a deterioration of the hysteresis regardless of bias polarity, but at a lower rate under positive bias.

### 3.4. Field Detection 

**Figure 5:**
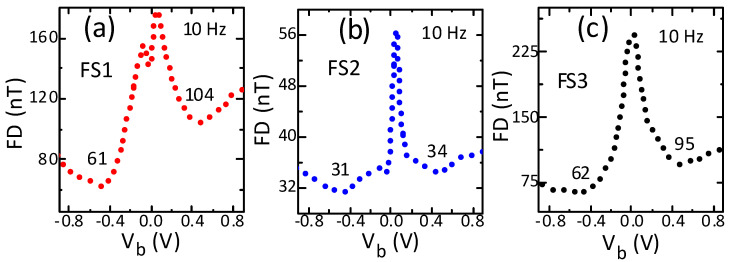
Influence of bias voltage on low-frequency field detection of sensors with high (**a**), medium (**b**) and low (**c**) sensitivity. The field detection was measured at the highest sensitivity point on the transfer curve. The field detection performance showed significant improvement with bias and all sensors showed the best field detection under negative polarity.

The low-frequency field detection of all sensors improved (declined) strongly with bias, reaching the lowest value for a specific bias and under negative polarity (Figure 5a–c). The low-frequency field detection declined with bias of both polarities by a factor of 1.1 to 3.9. The reduction was higher under negative bias and the highest (3.9) for sensor FS3. Importantly, all sensors showed the minimum field detection under both polarities for the same magnitude of 0.5 V. For all sensors, the minimum field detection was lower under negative bias. The minimum field detection differed most significantly (by a factor of 1.7) between the two polarities again for sensor FS3. In contrast to nonlinearity and hysteresis, the minimum field detection (31 nT) was shown in neither the highest field sensitivity sensors (FS1) nor the lowest field sensitivity sensors (FS3), but in the medium sensitivity sensors (FS2). This is consistent with the previously reported results [7] and implies that the increase in field sensitivity with bias was higher than that of the low-frequency noise. Moreover, it reached its minimum (best) value under a certain magnitude and negative polarity. However, the bias magnitude was not the one for which the sensitivity was maximum. These results indicate an important and significant role of bias magnitude and polarity in improving field detection performance.

**Figure 6:**
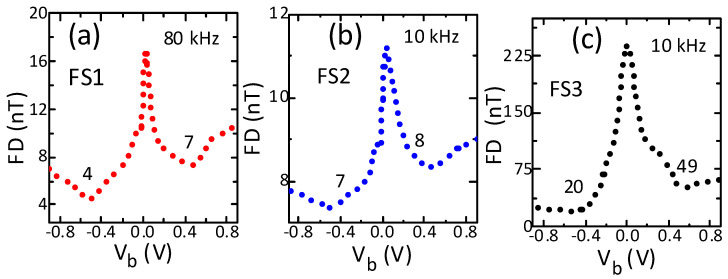
Influence of bias voltage on high-frequency field detection of sensors with high (**a**), medium (**b**) and low (**c**) sensitivity. The field detection was measured at the highest sensitivity point on the transfer curve. The field detection showed significant improvement with bias and all sensors showed the best field detection under negative polarity.

The high-frequency field detection of the sensors also improved significantly with bias, but the polarity influence was significant only for sensor FS3 (Figure 6a–c). The reduction in the high-frequency field detection with bias was similar to that observed for low-frequency sensors, except for the sensor FS3, which was significantly higher by a factor of five under positive bias and by a factor of 12 under negative bias. Again, all sensors showed the minimum field detection under both polarities for the same bias (0.5 V), as with the low-frequency field detection results. The minimum field detection was lower under negative bias for all sensors and it most significantly differed (by a factor of 2.4) between the polarities for sensor FS3. Interestingly, the high-frequency field detection was the lowest again for the same sensor (FS2) as in the low-frequency field detection experiment. The similar dependence of high- and low-frequency field detection—both showing the minimum value for the same bias magnitude and polarity—emphasizes the importance and significance of the bias magnitude and polarity in improving both low- and high-frequency field detection performance.

## 4. Conclusions

This paper investigated the influence of the polarity and magnitude of the bias voltage on the fundamental sensing characteristics of CoFeB/MgO/CoFeB-based tunneling magnetoresistance sensors. We investigated the field sensitivity, nonlinearity hysteresis and field detection. The field sensitivity, nonlinearity and hysteresis were extracted from current versus magnetic field curves and field detection was measured directly. For the investigation we fabricated sensors with the same properties except for the field sensitivity, which ranged from 2.7 V/T to 127 V/T.

All sensors showed a rapid increase (by a factor of 17 to 32) in field sensitivity with bias up to a significant magnitude. The increase rate and maximum values were higher under negative polarity. Moreover, the increase was linear up to significant voltage magnitudes. These behaviors indicate that both the magnitude and polarity of the bias voltage are important factors in improving and controlling the TMR sensors sensitivity.

In contrast to the sensitivity, the nonlinearity deteriorated (increased) with the bias magnitude. It changed from minimum to maximum by a factor of 10 to 26. The increase was linear and at higher rate for negative polarity than for positive polarity. The behavior of nonlinearity indicates the possibility of its reduction by selecting the proper bias polarity and linear control based on the bias voltage. The hysteresis of all sensors showed a linear increase with the bias for both polarities. However, under negative bias it was saturated and increased faster, reaching higher maximal values. These results imply that the choice of the bias voltage magnitude and polarity significantly affect the hysteresis of the sensor.

For all sensors, the detection of low- and high-frequency fields improved strongly with bias voltage, reaching the lowest value for a specific voltage magnitude and under negative polarity. However, the bias magnitude was not the factor for which the sensitivity reached the maximal values. The significant, linear and simultaneous effect of the improvement and deterioration of the sensing characteristics with bias voltage indicates that both bias magnitude and polarity are key factors in controlling and modifying sensing characteristics.

Our sensor design was based on perpendicular anisotropy in the sensing layer as a method for the linearization of the transfer curve. Therefore, future research is needed to confirm the results for other TMR sensors that are based on other designs for linearization, such as external field biasing, weakly pinned sensing layers and superparamagnetic sensing layers [2,22].

## Data Availability

Not applicable.
